# Pus Dyscrasia

**Published:** 1881-01

**Authors:** W. C. Barrett

**Affiliations:** Buffalo, N. Y.; President of the American Dental Association, etc.


ARTICLE IV.

### PUS DYSCRASIA.

BY W. C. BARRETT, M. D., D. D. S. BUFFALO, N. Y.

President of the American Dental Association, etc.

The origin and composition of pus has long been a question of more or less excited controversy among pathologists, and it has not as yet been made clear to the comprehension of all. To me it seems closely connected with the functions of the spleen, and with the functions and office of the leucocytes of the blood. Its etiology is of peculiar importance to the dental practitioner, as involving the propriety of and necessity for constitutional treatment for certain so-called local lesions which are daily met in his practice.

In general terms pus may be defined as dead blood, as from this fluid it mainly has its origin. Under the microscope it presents the appearance of white rounded corpuscles, from 1.2500 to 1.3500 of an inch in diameter, floating in a colorless fluid, in which may also be found albumen, mucus, &c., &c. That this fluid is the extravasated serum or liquor sanguinis of the blood admits of no dispute; but whence comes the pus corpuscle? The microscope reveals no difference between it, the white corpuscle of the blood, and the wandering leucocyte described by Virchow and other observers. That they are positively identical cannot be indisputably maintained, because no one has as yet succeeded in tracing the white corpuscle of the blood to its extrusion as a pus globule, but that they are identical seems probable from the fact that they cannot be distinguished from each other, except by their location. Rindfleisch positively asserts that the chief mass of pus is everywhere formed by the immigration of colorless blood corpuscles, but he does not clearly demonstrate it. Virchow and Bilrath declare that these are indistinguishable from genuine leucocytes, those wandering bodies which, through their amorphorus condition, may penetrate any tissue, and most pathologists believe them identical. We shall then take it for granted that the pus corpuscle is the leucocyte, deprived of its vitality and its power to act in the organization of tissue. How is it that we find it exuded? There are certain systemic conditions in which the white blood corpuscles exist in the blood in large numbers. Their average normal numerical proportion to the red discs is about as 1 to 450, but after the ingestion of a hearty meal this proportion is somewhat increased. In some cachetic conditions the white corpuscles are increased in number until they form one-tenth of the whole, and they then give a changed resemblance to the blood, and in certain forms of lukæmia they may even become equal in number with the red corpuscles, and impart to that fluid a decidedly milky appearance. In the blood of the splenic vein the number of white discs exceeds that in the artery from five to ten times, so that in the passage of the blood through the spleen the white corpuscles have been greatly increased in number, and it is also considered an established fact that the lymph glands have something to do with their formation, and this increase, in both the spleen and the lymphatics, is normally greatest after the ingestion of food. An examination of the spleen at such times, Rindfleisch says, shows the malphigian bodies distinctly tumified, as is also the case with the parenchyma of the lymph glands, and this is due to the temporary hyperæmia of those organs. Within their tissues may be found many newly formed cells mingled with the lymph. leukæmia, or leucocythæmia is readily found associated with enlarged lymph glands; and a large majority of the cases are accompanied with a tumid spleen. We may then trace leukæmia, or the abnormal disproportion between the white and red corpuscles, directly to those organs which seem to form the genesis of the colorless corpuscles of the blood.

If, as seems probable, the white corpuscles or leucocytes are the agents used in the restoration of lost tissue, if it be their office to organize protoplasm into elementary fibre, then any undue irritation or inflammation of a part will have a tendency to attach them to itself, and it is a fact that upon applying irritating agents to tissues which may in the life of the animal be brought under the observation of the microscope, these wandering cells have been seen to leave the blood-vessels and migrate to the neighboring tissues. During the condition of leukæmia, when the leucocytes are inordinately multiplied; great quantities of them, under the local morbidity of any inflammation, may thus be projected into surrounding tissues. Here, under the pathological conditions, the corpuscles may lose their vitality and be exuded as pus cells, mingled with the plasma or liquor sanguinis which is also extravasated through osmosis, and thus pus may be formed, and as the white corpuscles multiply in number the pus is increased in volume. When these pus corpuscles undergo a change and putrefy, the pus becomes sanious, and when the surrounding tissue is involved by a yet further retrogradation it is ichorous, and this may increase until sphacelus is present in all of the affected organs. Then it may be seen that a leukæmic condition may give rise to a pus dyscrasia, and that the continuance of the latter is dependent upon the former.

As dentists we are well aware of the fact that there are patients, the pulps of whose teeth are no sooner devitalized, with, of course, more or less of local irritation, than signs of incipient alveolar abscess present themselves, and despite every prevention or remedial measures, the disease steadily progresses to suppuration, which, once established, it is exceedingly difficult, nay, in some instances impossible, to cure without the complete removal of the tooth, which seems to be the irritating agent. There is such a cachetic condition that any comparatively slight irritation results in the extrusion of an inordinate amount of pusulent matter, and this I have called the pus dyscrasia. That it may be the result of an undue proportion of white corpuscles in the blood, and that its cure may be found in the usual treatment for leucocythæmia, seems highly probable, to say the least, and quite worthy the careful investigation of every student in pathology. What that treatment should be, or any account of other changes in the blood, which accompany this peculiar diathesis, it is not the object of this article to consider.

