# Correction from W. C. Barrett

**Published:** 1881-02

**Authors:** 


					﻿Editor of the hidependent Practitioner-
Dear Sir :—In the January No. of your excellent Journal
beneath my name at the head of an article entitled Pus
Dyscrasia, I am mentioned as “ President of the American
Dental Association.” Will you kindly say in the next number
that the line was not in my manuscript, and that I have no
desire to assume titles nor wear honors to which I have not
the shadow of a right. Prof. C. N. Peirce, of Philadelphia,
is the honored President of^ the American Dental Association,
and I would not have him or any one else think that I am
masquerading in stolen plumes.
Most truly yours,	W. C. Barrett.
Buffalo, N. Y., Jan. 28, 1881.
Rest assured, dear doétors Barrett and Peirce, that the .error
above referred to, which we cheerfully correct, is a mistake of
my mind, not soul. Dr. Barrett is 1st Vice-President. B. M. W.
				

